# Expanding the Spectrum of Oculocutaneous Albinism: Does Isolated Foveal Hypoplasia Really Exist?

**DOI:** 10.3390/ijms23147825

**Published:** 2022-07-15

**Authors:** Camilla Rocca, Lucia Tiberi, Sara Bargiacchi, Viviana Palazzo, Samuela Landini, Elisa Marziali, Roberto Caputo, Francesca Tinelli, Viviana Marchi, Alessandro Benedetto, Angelica Pagliazzi, Giacomo Maria Bacci

**Affiliations:** 1 Department of Biomedical Experimental and Clinical Sciences “Mario Serio”, University of Florence, 50121 Florence, Italy; c.rocca@unifi.it (C.R.); lucia.tiberi@unifi.it (L.T.); 2 Medical Genetics Unit, Meyer University Hospital, 50139 Florence, Italy; sara.bargiacchi@meyer.it (S.B.); viviana.palazzo@meyer.it (V.P.); samuela.landini@meyer.it (S.L.); pagliazzi.angelica@gmail.com (A.P.); 3 Pediatric Ophthalmology Unit, Children’s Hospital A, Meyer-University of Florence, 50139 Florence, Italy; elisa.marziali@meyer.it (E.M.); roberto.caputo@meyer.it (R.C.); 4 Department of Developmental Neuroscience, IRCCS Stella Maris Foundation, 56128 Pisa, Italy; francesca.tinelli@fsm.unipi.it (F.T.); viviana.marchi@fsm.unipi.it (V.M.); benedetto.alessandro@gmail.com (A.B.)

**Keywords:** foveal hypoplasia, OCA, *TYR*, misrouting, VEP, Trios-WES, hypomorphic allele, good BCVA, albinism

## Abstract

Oculocutaneous albinism is an autosomal recessive disorder characterized by the presence of typical ocular features, such as foveal hypoplasia, iris translucency, hypopigmented fundus oculi and reduced pigmentation of skin and hair. Albino patients can show significant clinical variability; some individuals can present with only mild depigmentation and subtle ocular changes. Here, we provide a retrospective review of the standardized clinical charts of patients firstly addressed for evaluation of foveal hypoplasia and slightly subnormal visual acuity, whose diagnosis of albinism was achieved only after extensive phenotypic and genotypic characterization. Our report corroborates the pathogenicity of the two common *TYR* polymorphisms p.(Arg402Gln) and p.(Ser192Tyr) when both are located *in trans* with a pathogenic *TYR* variant and aims to expand the phenotypic spectrum of albinism in order to increase the detection rate of the albino phenotype. Our data also suggest that isolated foveal hypoplasia should be considered a clinical sign instead of a definitive diagnosis of an isolated clinical entity, and we recommend deep phenotypic and molecular characterization in such patients to achieve a proper diagnosis.

## 1. Introduction

Oculocutaneous albinism (OCA) is a heterogeneous autosomal recessive disorder caused by the complete absence or reduction of melanin biosynthesis in melanocytes. OCA’s main clinical features concern the eyes, epidermis and hair, while ocular albinism (OA), an X-linked disorder caused by variants in the *GPR143* gene, only determines ocular manifestation [1].

Oculocutaneous albinism can occur in syndromic and non-syndromic forms; in the latter, the typical OCA features coexist with other and more severe abnormalities, as in Hermansky–Pudlak and Chediak–Higashi syndromes. To date, 20 genes have been identified as causative for the different clinical presentations of albinism, and eight of them are responsible for the non-syndromic forms. Mutation in *TYR*, *OCA2*, *TYRP1* and *SLC45A2* genes are the main cause of oculocutaneous albinism, and the respective pathologies are named with the acronyms OCA types 1 (A and B) to 4. Recently, one new gene, *DCT*, was identified as causative for OCA type 8 [2].

Albinos’ phenotype is widely variable and, according to the type of albinism, it can vary from a complete absence of pigmentation in hair, skin and eyes to a mild depigmentation. All types of OCA are described as having similar ocular features, which may include nystagmus, iris transillumination defects, fundus hypopigmentation, foveal hypoplasia, reduced visual acuity (usually in the range of 0.5 to 1.3 LogMAR), photophobia and sometimes a degree of color vision impairment. A peculiar finding, the absence of which excludes the diagnosis of albinism, is misrouting of the visual pathways, characterized by excessive crossing of fibers in the optic chiasma, which can result in strabismus and reduced stereoscopic vision [3,4].

In the absence of molecular testing, it is sometimes difficult to distinguish albinism from other conditions such as idiopathic infantile nystagmus, *SLC38A8*-related FHONDA syndrome and isolated foveal hypoplasia. In this regard, Kruijt et al. proposed major and minor diagnostic criteria for albinism. Major criteria include foveal hypoplasia of grade 2 or higher, optic nerve misrouting and ocular hypopigmentation and either iris translucency or fundus hypopigmentation of grade 2 or higher; minor criteria include nystagmus, hypopigmentation of skin and hair, grade 1 fundus hypopigmentation and grade 1 foveal hypoplasia. They proposed three major criteria or two major and two minor criteria to be necessary for clinical diagnosis, while, in the presence of a molecular diagnosis, one major criterion or two minor criteria should be sufficient [5,6].

Recently, many studies highlighted a role for a complex *TYR* genotype in determining a mild form of oculocutaneous albinism: a double-polymorphism haplotype, p.[Ser192Tyr;Arg402Gln], existing on the *trans* allele to a pathogenic *TYR* variant [5,7,8,9,10].

The aim of this study was to report unexpected findings in a series of patients who have bilateral foveal hypoplasia and mild visual acuity reduction. In addition, the study included patients for whom the diagnosis of albinism was given only after careful genotypic and phenotypic characterization, including a search for visual pathways misrouting, due to an extremely mild clinical presentation.

## 2. Results

### 2.1. Clinical Findings

We identified eight patients with a mean age at examination of 16.3 years (range 8–39). All but one (P8 is the father of P7) patient were paediatric: three female (37.5%) and five male (62.5%) patients.

**Figure 1:**
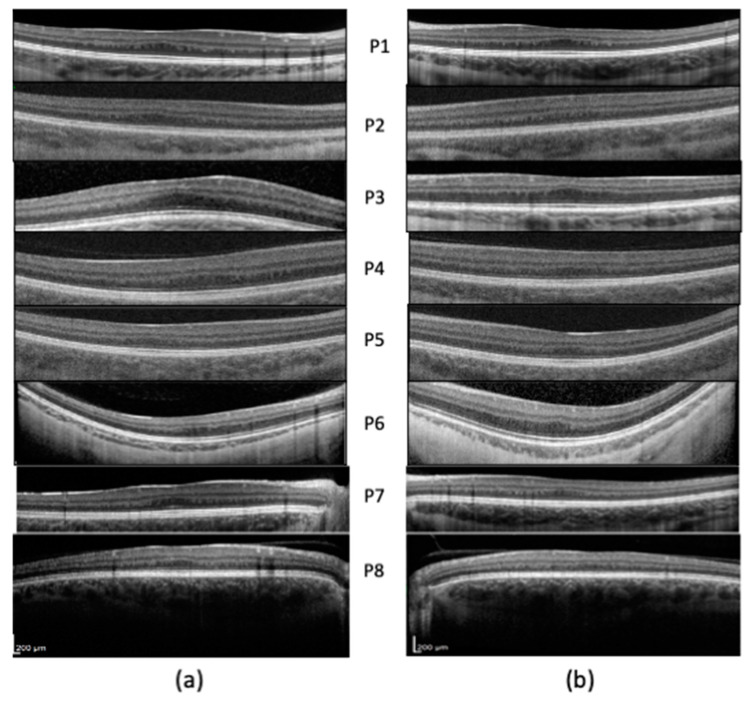
Structural SD-OCT examination: note the different degrees of foveal hypoplasia (refer to Table 1 for grading). (**a**) Right eye. (**b**) Left eye.

**Figure 2:**
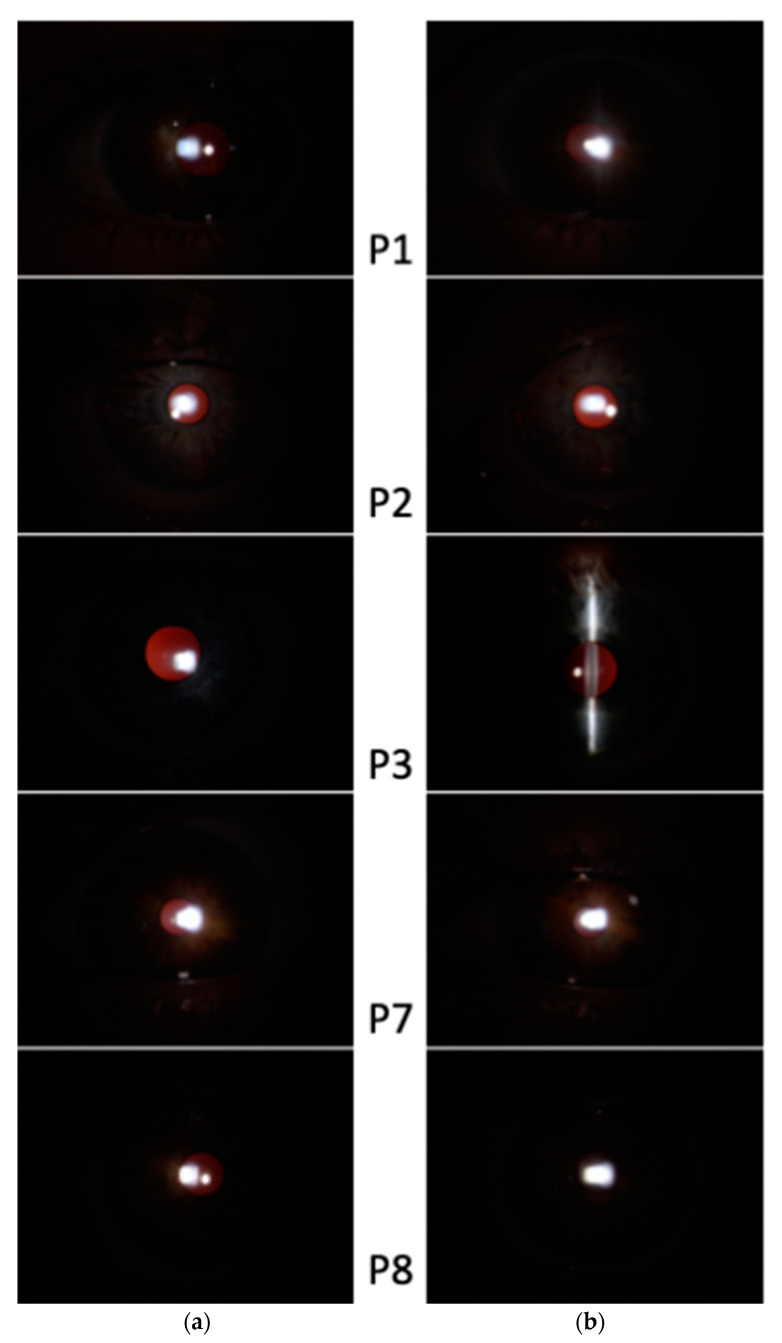
Slit-lamp examination: note the absence of iris translucency on retro-illumination. (**a**) Right eye. (**b**) Left eye; (images acquired with 10× magnification).

**Figure 3:**
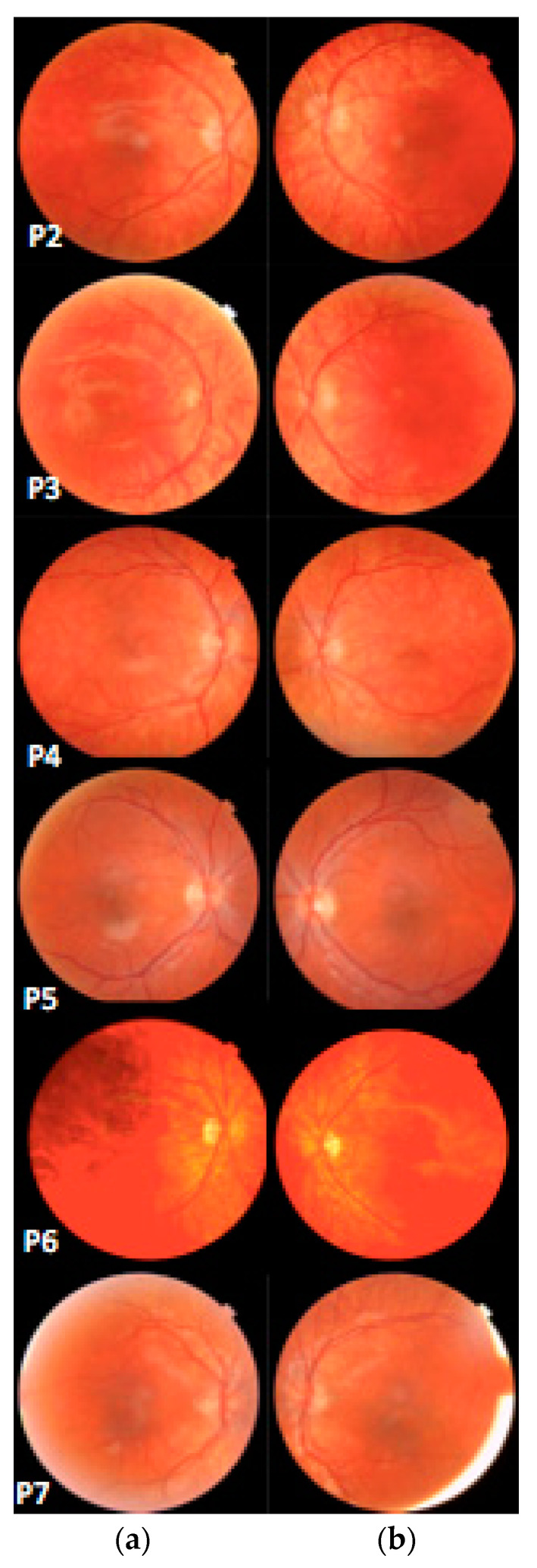
Fundus photography showing the degree of retinal hypopigmentation according to Krujit classification. (**a**) Right eye. (**b**) Left eye; (45° wide angle acquisition).

The mean best corrected visual acuity (BCVA) was 0.20 LogMAR (range 0–0.3 LogMAR). All patients had hypoplastic foveal pits; five out of eight patients (10 eyes; 62.5%) presented a grade 2 foveal hypoplasia, whereas three out of eight patients (six eyes; 37.5%) had a grade 3 foveal hypoplasia (Figure 1). At fundus examination, five out of eight patients (10 eyes; 62.5%) presented mild retinal hypopigmentation, while three out of eight patients (six eyes; 37.5%) had normal retinal pigmentation (Figure 2). No patients presented nystagmus, while four out of eight patients (eight eyes; 50.0%) presented different forms of strabismus. Iris transillumination defects were absent in all patients (Figure 3). The clinical features of the patients are summarized in Table 1.

### 2.2. Molecular Analysis

Trios whole-exome sequencing (WES) was performed in our series of eight patients from six unrelated families; identified variants were prioritized as described in Materials and Methods and in agreement with American College of Medical Genetics and Genomics (ACMG) guidelines. Molecular analysis showed point mutations in *TYR* in all patients analyzed, and none of them presented sequence alterations or copy number variants (CNVs) in *PAX6*, *SLC38A8* or in other genes associated with foveal hypoplasia. Among all mutations, we identified six missense and one frameshift; only variant p.(Pro152Arg) (cases 7–8) was not previously reported in the literature. All variants identified were classified as pathogenic in accordance with the ACMG. A complex, known *TYR* haplotype, composed by the hypomorphic allele p.[Ser192Tyr;Arg402Gln], was identified in seven out of eight patients (cases 1, 2 and 4–8) *in trans* with a pathogenic *TYR* variant [5,9]. In one family, segregation analysis led to cascade diagnosis in the father (P7, P8) who had the same mild clinical manifestations; in both cases, the pathogenic variant p.(Pro152Arg) was found *in trans* with the hypomorphic allele p.[Ser192Tyr;Arg402Gln]). Genetic analysis revealed compound heterozygous variants p.[Met96Asnfs*73];[Gly109Arg] only in patient 3, in the *TYR* gene, in the absence of hypomorphic alleles; both variants were previously described as being associated with oculocutaneous albinism [11,12]. In two sisters (cases 4–5), segregation analysis was not able to establish the *cis*/*trans* position of the variants because p.(Arg402Gln) was present in a heterozygous state in both parents. To further support our hypothesis that the two common polymorphisms were *in cis* in the two sisters, in this family, we genotyped nine highly polymorphic microsatellite markers in the regions flanking the *TYR* gene (chr11:85907157-90375771); this allowed us to show that the two sisters were haplo-identical in this chromosomal region (Supplementary Table S1). These results, together with the clinical phenotype, suggest that patients 4 and 5 inherited the two common polymorphisms *in cis* from the same parent, and both are located on the *trans* allele of the pathogenic *TYR* variant p.(Gly47Asp) [13].

Genotype findings for each study participant are summarized in Table 2 (see Figure A2 and Table A1 in Appendix B for family pedigrees and detailed variant information).

### 2.3. Visual Evoked Potentials (VEPs)

**Figure 4:**
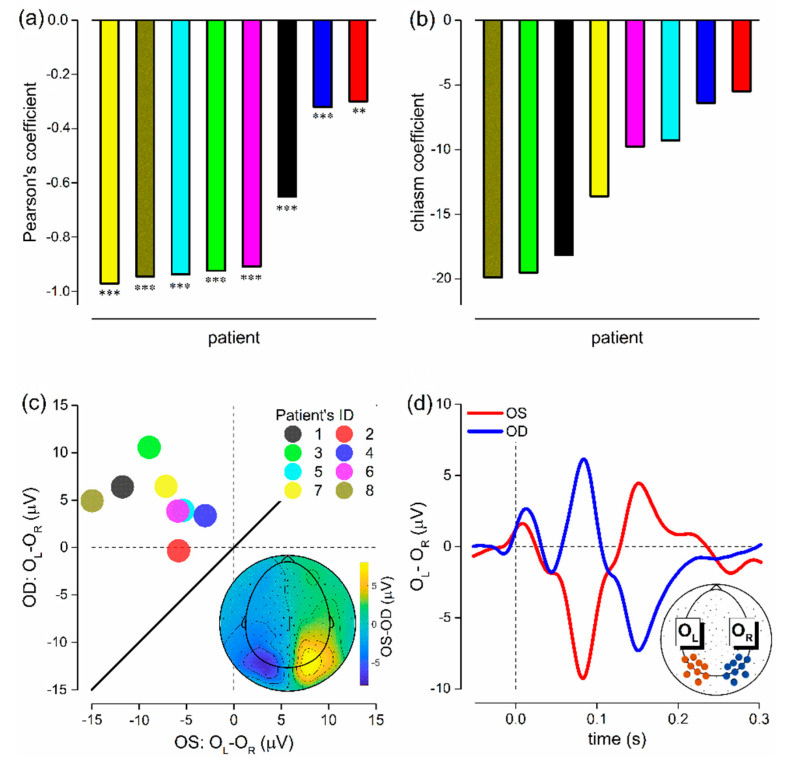
Misrouting of the optic pathway. (**a**) Pearson’s coefficient of correlation computed between the OS and OD mean differential activity (OL-OR, from 0.07 to 0.3 s). Each bar represents a patient (color codes for patients is consistent across panels, see panel c). The within-subject correlation was statistically significant for all patients (*p* ≤ 0.0011. Asterisks mark the statistical significance: *** < 0.001, ** < 0.01). (**b**) Chiasm coefficient computed between the OS and OD mean differential activity (OL-OR, from 0.07 to 0.1 s). Each bar represents a patient. (**c**) Scatter plot showing the average differential activity for both OS and OD. Each dot represents a patient. All the dots scattered above the equality line (thick, black) indicate the presence of a significant interhemispheric difference (*p* > 0.001). The topoplot inset shows the grand-average difference between OS and OD from 0.07 to 0.1 s, confirming the presence of an interhemispheric difference mostly expressed over occipital electrodes. (**d**) Grand-average differential (OL-OR) activity for OS (red) and OD (blue). The inset reports the position of two sets of electrodes of interest (OL in dark red, OR in dark blue).

**Figure A1:**
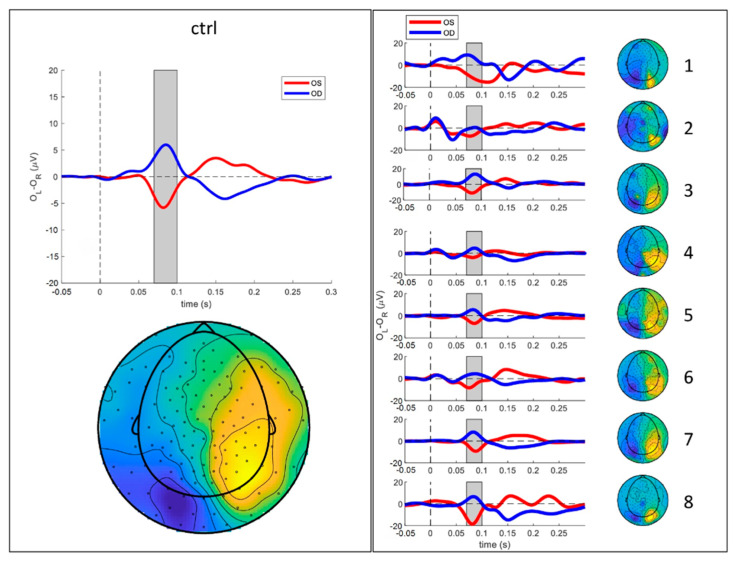
Single-subject misrouting effects. **Left** panel shows the typical interhemispheric asymmetry for a control albino patient. The plot shows the differential (O_L_–O_R_) activity for OS (red) and OD (blue). The lower topoplot shows the difference between OS and OD from 0.07 to 0.1 s (grey area), confirming the presence of an interhemispheric difference mostly expressed over occipital electrodes. **Right** panel is the same as the left panel but for each patient from the current study.

**Figure A2:**
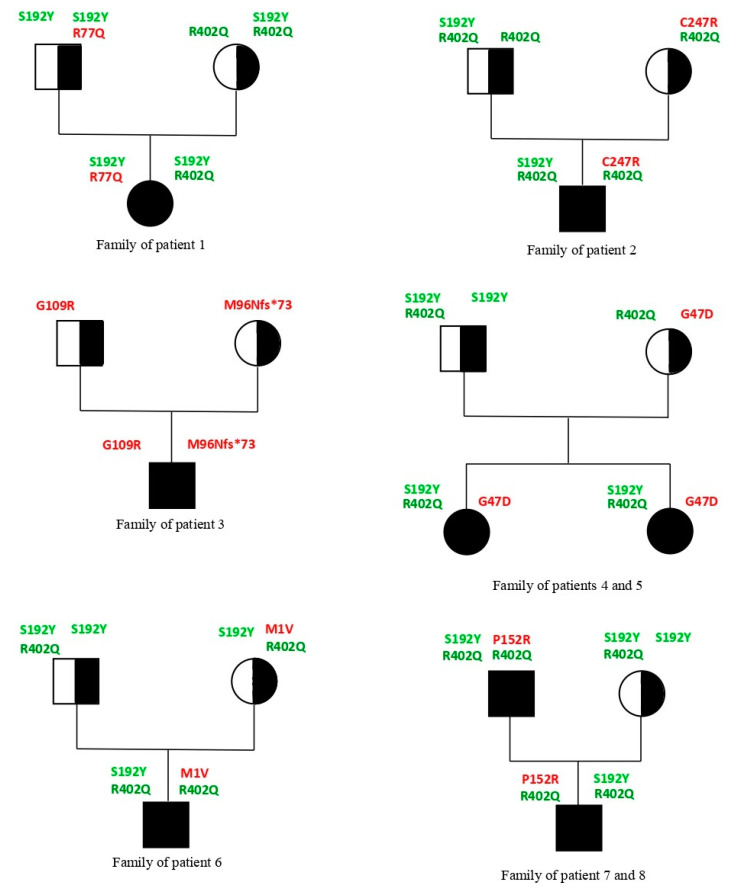
Family pedigrees (in red *TYR* pathogenic variants, in light green the p.(Ser192Tyr) polymorphism and in dark green the p.(Arg402Gln)polymorphism).

Figure 4 summarizes the results from the visual evoked potential experiment. All patients (N = 8) revealed a significant (*p* ≤ 0.0011), negative correlation between the differential EEG responses evoked under the OS and OD visual stimulation (Figure 4a), as well as a negative chiasm coefficient (Figure 4b). A two-tailed, paired *t*-test on the chiasm coefficient confirmed the presence of generalized signal reduction for the ipsilateral hemisphere (t(7) = −6.1607, *p* > 0.001, see Figure 4c), mostly expressed over the occipital region (inset in Figure 4c). Consistent with the literature [21,22,23,24], this hemispheric asymmetry was mostly visible from about 50 to 200 ms from stimulus onset (Figure 4c. See Figure A1 in Appendix A for single-subject evoked responses). Overall, these results demonstrated in all patients the presence of a misrouting of the optic pathway.

## 3. Discussion

Foveal hypoplasia with a substantial reduction in BCVA, usually in the range of 0.5 to 1.3 LogMAR, is a typical clinical feature in oculocutaneous and ocular albinism. In the albino phenotype, other distinctive ocular changes include nystagmus, reduced iris pigmentation with iris translucency, reduced retinal pigmentation, misrouting of the optic nerve fibers at the chiasm, strabismus and reduced stereoscopic vision. To demonstrate selective misrouting, pattern-onset VEPs are usually performed with a technique specifically designed for this purpose; thus, a conventional, simultaneous binocular VEP is not able to demonstrate this anomaly. Normal routing of the optic pathways excludes the diagnosis of albinism [3]. The VEP is normally not necessary for the diagnosis of albinism because misrouting is implied by the observation of a typical ocular phenotype. In some patients with mild hypopigmentation (a few with OCA1B), foveal hypoplasia and no obvious nystagmus, a VEP may be a useful adjunct to demonstrate misrouting of the retinal to occipital projections [25,26]. MRI studies may demonstrate misrouting, but this approach is not validated sufficiently to replace VEP [27].

In this paper, we retrospectively reviewed the clinical charts of a series of patients first referred to the Ocular Genetic Service for a clinical suspect of isolated foveal hypoplasia associated with a bilateral, moderate decrease in BCVA. None of these patients presented nystagmus or iris translucency, whilst all of them showed a misrouting of optic pathways. By naked-eye observation, no obvious whitening phenomenon in the patients’ skin, hair or eyes was observed, although, in all cases, skin and hair pigmentation were lighter than their parents’. In all patients, deep phenotyping was performed to definitely assess morphological features to correlate with molecular data analysis.

For molecular analysis, as previously stated, an in silico gene panel composed by all known genes associated with foveal hypoplasia and other related conditions was selected. In all analyzed patients, molecular analysis showed mutations in the *TYR* gene, which is known to be responsible for type I OCA [28]. In all our patients, segregation analysis demonstrated that the identified genotypes segregated with phenotype and, therefore, helped us to get to a definitive diagnosis.

As previously reported, in patients 1, 2 and 4–8, a pathogenic *TYR* variant (different for each family) was detected *in trans* with both the two common *TYR* polymorphisms p.[Ser192Tyr;Arg402Gln]. There has been a long, ongoing debate about the two common *TYR* polymorphisms p.(Ser192Tyr) and p.(Arg402Gln) and their role in determining albinism when they are both *in trans* with a pathogenic, inactivating mutation [5,7,8,9].

Those two polymorphisms are common in the general population, with allele frequencies of 25.4% and 17.6%, respectively (gnomAD v2.1.1), and are normally considered benign. The most recent evidence on the issue comes from Lin et al. in 2022; in their paper, the authors describe a big, consanguineous, Amish family in which the combination of the complex haplotype p.[Ser192Tyr;Arg402Gln] *in trans* with a pathogenic *TYR* variant segregated in all members with a mild albinism phenotype. They also described five members of this family with a fully penetrant mild clinical form of OCA who were homozygous for both the common *TYR* polymorphisms [10]. Molecular results in our series of patients confirmed the pathogenicity of this genotype, so we definitely agree with Lin et al. that the *TYR* p.[Ser192Tyr;Arg402Gln] haplotype should be included as a pathogenic allele and looked for in genetic diagnoses of albino patients. Moreover, we highlight that this specific genotype can be found in individuals with foveal hypoplasia without an obvious oculocutaneous albinism phenotype; our series of patients indeed received an OCA diagnosis only after performing VEP and molecular analysis. To further confirm the pathogenicity of this complex haplotype, we performed a comparison of the molecular results between our group of patients and our in-house Trios-WES control cohort (1000); no individual in our control cohort showed the simultaneous presence of a *TYR* pathogenic variant and the complex haplotype p.[Ser192Tyr;Arg402Gln].

Furthermore, in proband 5, we identified two already known pathogenic variants in *TYR*, p.(Met96Asnfs*73) and p.(Gly109Arg), in a compound, heterozygous state [11,12]. Both variants have already been described in association with OCA type I; the phenotype-genotype comparison allowed us to make a diagnosis of a mild form of OCA type IB in this patient. Indeed, mutations completely abolishing tyrosinase activity result in OCA1A, the most compromised albinism phenotype, while mutations allowing some enzyme activity result in OCA1B, a milder phenotype that permits some accumulation of melanin pigment [3].

Previous to this study, there were very few reports of *TYR* mutations in patients with such a mild albino phenotype, presenting only foveal hypoplasia as a main clinical feature; Kubal et al., in 2009, identified compound heterozygous variants in the *TYR* gene in a case of foveal hypoplasia, in which the patient showed only mild ocular features without apparent skin or hair whitening, nystagmus, photophobia or severely reduced vision [29]; and Xu et al., in 2020, identified compound, heterozygous *TYR* variants using a WES approach in another patient with isolated foveal hypoplasia. Xu complained that, due to cases of isolated foveal hypoplasia being exceedingly rare, they could not study more samples to support the pathogenicity of their results [30].

In this study, we were able to characterize eight patients presenting with foveal hypoplasia and misrouting of the optic nerves with a *TYR* genotype constituted by a pathogenic, inactivating mutation and the complex allele p.[Ser192Tyr;Arg402Gln] *in trans*. We believe that these results strongly support the role of *TYR* and the complex allele p.[Ser192Tyr;Arg402Gln] in determining an extremely mild form of OCA that, without performing VEP or molecular analysis, could be overlooked. We hypothesize the possibility that patients with moderately decreased bilateral visual acuity and a degree of foveal hypoplasia could mask a larger group of albino phenotypes that can be easily overlooked if proper genotyping is not performed. 

Moreover, the added value of this paper is the suggestion that the presence of an apparently isolated foveal hypoplasia must be considered cautiously since it is only a clinical sign instead of a definite diagnosis of an isolated clinical entity; in fact, the series described the association of foveal hypoplasia with a misrouting of optic pathways in agreement with a less-than-mild albino phenotype.

Finally, it is a fact that the reported haplotype has been recently associated with the albino phenotype [5,9,10], but, unlike FHONDA [31] and the different known forms of albinism, the striking data of our series rely on a good, retained BCVA in all patients; to the best of our knowledge, this is the first paper that reports albino phenotypes with such good functional preservation. Our report shows that this specific genotype can also cause an uncommonly mild form of albinism characterized solely by foveal hypoplasia, a moderate decrease in visual acuity and misrouting of the optic pathways. 

In conclusion, our hypothesis is that the real spectrum of albinism must be broadened, and albinism diagnosis should always be considered in patients with foveal hypoplasia who did not obtain a molecular definition.

## 4. Materials and Methods

### 4.1. Clinical Evaluation

Between April and May 2022, we retrospectively reviewed standardized clinical charts of patients referred to the Rare Eye Diseases Clinic of Meyer Children’s Hospital, Florence (Italy). Inclusion criteria were the presence of foveal hypoplasia (FH), optic pathways misrouting and BCVA better than 0.5 LogMAR. All patients included in the study were genetically characterized.

All patients underwent a complete, standard ophthalmological examination including: age at last examination, best corrected visual acuity (BCVA) using age-appropriate ETDRS charts with LogMAR conversion, slit-lamp examination of anterior segment (with retro-illumination technique to demonstrate iris translucency), indirect fundoscopic examination, ocular motility assessment and color vision evaluation. The past medical and familiar history of patients were also taken into consideration.

Moreover, all patients underwent spectral domain optical coherence tomography (SD-OCT) either with the Spectralis platform (Heidelberg Engineering GmbH, Heidelberg, Germany), iVue system (Optovue Inc, Fremont, CA, USA, ver.3.3.0.42.) or Topcon 3D, DRI OCT Triton, (Topcon Corporation, Tokyo, Japan), in addition to pattern-onset VEPs (see detailed description in 4.2 subsection) and fundus photography (TRC-NW400, Topcon Medical System, Inc., Oakland, NJ, USA).

The degree of FH was evaluated based on the cross-sectional OCT images collected and graded from 1 to 4 according to Thomas classification [32]. Fundus hypopigmentation was graded according to Krujit classification [6].

Informed consent was obtained from all patients’ guardians. The study protocol was conducted in conformity with the tenets of the Declaration of Helsinki, and it was approved by the local Ethics Committee of Meyer Children’s University Hospital.

### 4.2. Visual Evoked Potential Procedure

The visual stimuli to elicit the VEPs were generated with Psychtoolbox for Matlab (Matlab r2018b, The Mathworks, Inc., Natick, MA, USA) and displayed on a gamma-calibrated CRT monitor (resolution of 800 × 600 pixels, refresh rate of 60 Hz). The synchronization between the EEG activity and the visual stimulation was assured by means of a Ni-DAQ USB-6001.

EEG was recorded using a 128-channel HydroCell Sensor Net (Electrical Geodesics Inc.) with a sampling rate of 500 Hz. The scalp electrodes were positioned according to the 10–20 international system and referenced to an average reference.

The visual stimulus consisted of a high-contrast checkerboard square (side 28°, 98% contrast, square checks varying randomly within each session, being either 0.5°, 1° or 2°) with a stylized face displayed in red at the center of the stimulus, comprising three dots and a line (stylizing eyes, nose and mouth). Patients (N = 8) were asked to maintain fixation on the nose of the stylized face for the entire duration of the session. In about 5% of trials, a smile or a sad face appeared, and participants were asked to report the number of times the face smiled. Left (oculus sinister, OS) and right (oculus dexter, OD) eyes were stimulated monocularly in separate blocks at a viewing distance of 57 cm. The pattern-onset stimulation was presented as repeating blocks of 40 ms ON and 440 ms OFF (498 ± 51 and 498 ± 51 stimulations for OS and OR, respectively. Mean ± standard deviation).

Using the MATLAB toolbox EEGLAB in combination with the plugins firfilt, cleanline and clean_rawdata. The EEG data were referenced to CZ, noisy electrodes were removed and the signal high-pass filtered (cut-off of 0.1 Hz, Blackman sinc FIR filter with a transition bandwidth of 0.2 Hz); line noise was removed, artifacts were corrected by means of Artifact Subspace Reconstruction (ASR, burst criterion = 20) [33] and bad channels were interpolated. Trials were epoched relative to the stimulus presentation from −0.2 to 0.4 s and baseline corrected (baseline computed within −0.05 to 0 s). The VEPs were then low-pass filtered at 35 Hz with an IIR Butterworth filter from the MATLAB toolbox Fieldtrip. We, a priori, defined 9 electrodes of interest for the left (OL) and right (OR) hemisphere over the occipital pole (see Figure 4). The VEP was calculated for both OL and OR recorded from both the OS and OD recordings. Interhemispheric differences were estimated by computing the differential EEG activity (OL-OR) of OS and OD. We defined the chiasm coefficient (computed within 0.07 and 0.1 s) and the Pearson’s coefficient of correlation (computed within 0.07 and 0.3 s) as the mean differential activity and the correlation between OS and OD, respectively [24]. They are both indexes of interhemispheric differences, with negative values for both indexes being indicative of the presence of a misrouting of the optic pathway.

### 4.3. Molecular Findings

For the molecular study, WES analysis was performed on each proband and their respective parents. DNA specimens were made anonymous by the application of a numeric code. DNA was extracted from peripheral blood using QIAamp Mini Kit (QIAGEN^®^, Hilden, Germany). Libraries were constructed with enzymatic fragmentation followed by end repair, A-tailing, adapter ligation and library amplification. Libraries were hybridized to the whole-exome capture arrays (SeqCap EZ Exome v3, Nimblegen, Roche, Basel, Switzerland) and sequenced with NextSeq500/550 (Illumina Inc., San Diego, CA, USA). The reads were aligned with the human reference hg19 genome using Burrows–Wheeler Aligner (BWA) (10.1093/bioinformatics/btp324), mapped and analyzed with the IGV software (Integrative Genome Viewer, 2013, Broad Institute) [34]. Downstream alignment processing was performed with the Genome Analysis Toolkit Unified Genotyper Module (GATK) [35], SAMtool [36] and Picard Tools (http://picard.sourceforge.net/ accessed on 14 December 2021). Variants were annotated using Annovar tool [37] to obtain information such as variant frequency in different populations and the predictions of the variant effect using different software (SIFT, Polyphen2, MutationTaster, MutationAssessor, FATHMM and FATHMM MKL). Quality control of sequencing showed that 96% of the reads were mapped to the reference genome (hg19), and 97% of the targeted regions were covered by ≧ 30× reads with average depth of 100×.

We retained non-synonymous, short insertion/deletion, synonymous or splice-site variants (20 bp splice acceptor, 20 bp splice donor) with the following characteristics:

- variants in genes associated with foveal hypoplasia reported in Online Mendelian Inheritance in Man (OMIM) and/or Human Gene Mutation Database (HGMD) or in the scientific literature revised on 30 April 2022;
- variants not present or with a minor allele frequency (MAF) ≤ 0.01 for autosomal recessive (AR) and with a MAF ≤ 0.001 for autosomal dominant (AD)-transmitted genes in population database “1000 Genomes Project”, “Exome Variant Server”, dbSNP153 and in our in-house exome control cohort (3000 exomes) of unrelated subjects analyzed for non-ocular diseases referred to the Medical Genetic Unit of the Meyer University Hospital (Florence, Italy);
- variants not present in the database of healthy control populations (gnomAD) in homozygous or hemizygous state;
- variants reported in disease-causing mutations databases as ClinVar, HGMD or predicted as damaging by at least 3 in silico tools (Polyphen-2, SIFT, Mutation Taster, MutationAssessor, FATHMM, FATHMM MKL). For splicing and synonymous variants, we retained only those predicted as causative of splicing alteration by the Berkeley Drosophila Genome Project (BDGP).

We used manual inspection for the p.(Arg402Gln) and p.(Ser192Tyr) variants in *TYR* [8]. In order to estimate copy number variations (CNVs), we used a normalized read count approach implemented *in house* [38].

Selected variants were classified in agreement with the interpretation guidelines of ACMG [39] as resulting from Varsome (https://varsome.com/ accessed on 30 April 2022), and we retained only those classified as “pathogenic”, “likely pathogenic” or “variant of unknown significance”.

All identified variants were confirmed by Sanger sequencing.

In patients 4 and 5 and relatives, nine already known microsatellite markers genetically mapped in the critical interval between D11S4197 and D11S1358 were analyzed (Supplementary Table S1). The STR markers were selected from UCSC database (https://genome.ucsc.edu/ accessed on 16 June 2022) and separated on 3500 genetic Analyzer (Waltham, MA, USA) using 600 LIZ size standard, and the data were analyzed by Gene Mapper software (Waltham, MA, USA).

## 5. Conclusions

This study, focused on a series of patients with a good, retained BCVA and only foveal hypoplasia as an evident clinical sign, suggests that the albino spectrum is probably wider than known up until today. It is a fact that so-called isolated foveal hypoplasia and mild oculocutaneous albinism are two clinical diagnoses that can sometimes be difficult to distinguish even with careful and expert evaluation. Some authors tried to provide clinical diagnostic criteria, but there are many situations in which even this approach cannot be resolutive.

We think that the most intriguing part of this paper relies on two main pillars:

1. First of all, we provided new support for the relevance of the complex haplotype p.[Ser192Tyr;Arg402Gln] in altering tyrosinase function and, so, determining *TYR* involvement in an extremely mild form of OCA;
2. Secondly, the albino genotype could be more prevalent than is reported in the literature.

The awareness of such phenotype variability should be emphasized among health professionals in order to give a higher detection rate of the albino phenotype and provide the basis for better clarification for developmental biology aiming to enable related therapies

## Figures and Tables

**Table 1:** Phenotypic data with a clinical distinction between major and minor criteria for albinism.

			Major Criteria						Minor Criteria	
			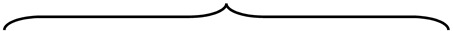						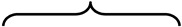	
ID	Sex	Age at Evaluation	Grading of FH	BCVA (LogMAR)		Iris Transillumination	Nystagmus	Misrouting	Strabismus	Fundus Pigmentation
				RE	LE					
**P1**	F	8	3	0.2	0.2	N	N	Y	Y	Grade 0
**P2**	M	12	3	0.2	0.3	N	N	Y	N	Grade 1
**P3**	M	13	2	0.3	0.3	N	N	Y	Y	Grade 1
**P4**	F	14	2	0.2	0.2	N	N	Y	N	Grade 1
**P5**	F	17	2	0.2	0.3	N	N	Y	N	Grade 1
**P6**	M	19	3	0.3	0.2	N	N	Y	N	Grade 1
**P7**	M	10	2	0.1	0.1	N	N	Y	Y	Grade 0
**P8**	M	39	2	0	0.1	N	N	Y	Y	Grade 0

BCVA: best corrected visual acuity; FH: foveal hypoplasia; Y: yes; N: no.

**Table 2:** Genetic findings.

ID	Family	Arg402Gln	Ser192Tyr	*TYR* Variant	References
Patient 1	Proband	Het	Hom	c.230G>A p.Arg77Gln	[14,15]
	Father	WT	Hom	c.230G>A p.Arg77Gln	
	Mother	Hom	Het	-	
Patient 2	Proband	Hom	Het	c.739T>C p.Cys247Arg	[16]
	Father	Hom	Het	-	
	Mother	Het	WT	c.739T>C p.Cys247Arg	
Patient 3	Proband	WT	WT	c.286dupA p.Met96Asnfs*73 c.325G>A p.Gly109Arg	[11,12,17]
	Father	WT	WT	c.325G>A p.Gly109Arg	
	Mother	WT	WT	c.286dupA p.Met96Asnfs*73	
Patients 4 and 5	Proband	Het	Het	c.140G>A p.Gly47Asp	[13,18]
	Sister	Het	Het	c.140G>A p.Gly47Asp	
	Father	Het	Hom	-	
	Mother	Het	WT	c.140G>A p.Gly47Asp	
Patient 6	Proband	Hom	Het	c.1A>G p.Met1Val	[19,20]
	Father	Het	Hom	-	
	Mother	Het	Het	c.1A>G p.Met1Val	
Patients 7 and 8	Proband	Hom	Het	c.455C>G p.Pro152Arg	-
	Father	Hom	Het	c.455C>G p.Pro152Arg	-
	Mother	Het	Hom	-	

Het: heterozygous, Hom: homozygous, WT: wild type.

## Data Availability

Not applicable.

## Supplementary Materials

The following are available online at https://www.mdpi.com/article/10.3390/ijms23147825/s1.

## Appendix B

**Table A1:** Detailed variant information.

gDNA	cDNA	Protein	dbSNP	GnomAD v2.1.1 (MAF General Population)	ACMG Criteria	ACMG Classification
chr11-88911351-G-A	c.230G>A	p.Arg77Gln	rs61753185	0.00007227	PM1, PM2, PM5, PP2, PP3, PP5	Pathogenic
chr11-88911860-T-C	c.739T>C	p.Cys247Arg	rs367543068	n.a.	PM1, PM2, PP2, PP3, PP5	Pathogenic
chr11-88911406-C-CA	c.286dupA	p.Met96Asnfs*73	rs61753190	n.a.	PVS1, PM2, PP5	Pathogenic
chr11-88911261-G-A	c.140G>A	p.Gly47Asp	rs61753180	0.0001556	PM1, PM2, PM5, PP2, PP3, PP5	Pathogenic
chr11-88911446-G-A	c.325G>A	p.Gly109Arg	rs61753253	0.00006366	PM1, PM2, PM5, PP2, PP3, PP5	Pathogenic
chr11-88911122-A-G	c.1A>G	p.Met1Val	rs28940881	0.00006718	PS1, PVS1, PM2, PP5	Pathogenic
chr11-88911576-C-G	c.455C>G	p.Pro152Arg	n.a.	n.a.	PM1, PM2, PM5, PP2	Likely Pathogenic

MAF: minor allele frequency; n.a. not available.

## References

[1] Schnur R.E., Gao M., Wick P.A., Keller M., Benke P.J., Edwards M., Grix A.W., Hockey A., Jung J.H., Kidd K.K. (1998). OA1 Mutations and Deletions in X-Linked Ocular Albinism. Am. J. Hum. Genet..

[2] Pennamen P., Tingaud-Sequeira A., Gazova I., Keighren M., McKie L., Marlin S., Halem S.G., Kaplan J., Delevoye C., Lacombe D. (2020). Dopachrome tautomerase variants in patients with oculocutaneous albinism. Genet. Med..

[3] Grønskov K., Ek J., Brondum-Nielsen K. (2007). Oculocutaneous albinism. Orphanet J. Rare Dis..

[4] Marçon C.R., Maia M. (2019). Albinism: Epidemiology, genetics, cutaneous characterization, psychosocial factors. An. Bras. Dermatol..

[5] Campbell P., Ellingford J.M., Parry N.R.A., Fletcher T., Ramsden S.C., Gale T., Hall G., Smith K., Kasperaviciute D., Thomas E. (2019). Clinical and genetic variability in children with partial albinism. Sci. Rep..

[6] Kruijt C.C., de Wit G.C., Bergen A.A., Florijn R.J., Schalij-Delfos N.E., van Genderen M.M. (2018). The Phenotypic Spectrum of Albinism. Ophthalmology.

[7] Jagirdar K., Smit D.J., Ainger S.A., Lee K.J., Brown D.L., Chapman B., Zhao Z.Z., Montgomery G.W., Martin N.G., Stow J.L. (2014). Molecular analysis of common polymorphisms within the human *Tyrosinase* locus and genetic association with pigmentation traits. Pigment Cell Melanoma Res..

[8] Norman C., O’Gorman L., Gibson J., Pengelly R., Baralle D., Ratnayaka J.A., Griffiths H., Rose-Zerilli M., Ranger M., Bunyan D. (2017). Identification of a functionally significant tri-allelic genotype in the Tyrosinase gene (TYR) causing hypomorphic oculocutaneous albinism (OCA1B). Sci. Rep..

[9] Grønskov K., Jespersgaard C., Bruun G.H., Harris P., Brøndum-Nielsen K., Andresen B.S., Rosenberg T. (2019). A pathogenic haplotype, common in Europeans, causes autosomal recessive albinism and uncovers missing heritability in OCA1. Sci. Rep..

[10] Lin S., Sanchez-Bretaño A., Leslie J.S., Williams K.B., Lee H., Thomas N.S., Callaway J., Deline J., Ratnayaka J.A., Baralle D. (2022). Evidence that the Ser192Tyr/Arg402Gln in cis Tyrosinase gene haplotype is a disease-causing allele in oculocutaneous albinism type 1B (OCA1B). npj Genom. Med..

[11] Khordadpoor-Deilamani F., Akbari M.T., Karimipoor M., Javadi G.R. (2016). Homozygosity mapping in albinism patients using a novel panel of 13 STR markers inside the nonsyndromic OCA genes: Introducing 5 novel mutations. J. Hum. Genet..

[12] Camand O., Marchant D., Boutboul S., Péquignot M., Odent S., Dollfus H., Sutherland J., Levin A., Menasche M., Marsac C. (2001). Mutation analysis of the tyrosinase gene in oculocutaneous albinism. Hum. Mutat..

[13] Oetting W.S., Witkop C.J., A Brown S., Colomer R., Fryer J.P., E Bloom K., A King R. (1993). A frequent tyrosinase gene mutation associated with type I-A (tyrosinase-negative) oculocutaneous albinism in Puerto Rico. Am. J. Hum. Genet..

[14] Kikuchi H., Hara S., Ishiguro S., Tamai M., Watanabe M. (1990). Detection of point mutation in the tyrosinase gene of a Japanese albino patient by a direct sequencing of amplified DNA. Qual. Life Res..

[15] Zhong Z., Gu L., Zheng X., Ma N., Wu Z., Duan J., Zhang J., Chen J. (2019). Comprehensive analysis of spectral distribution of a large cohort of Chinese patients with non-syndromic oculocutaneous albinism facilitates genetic diagnosis. Pigment Cell Melanoma Res..

[16] Urtatiz O., Sanabria D., Lattig M.C. (2014). Oculocutaneous albinism (OCA) in Colombia: First molecular screening of the TYR and OCA2 genes in South America. J. Dermatol. Sci..

[17] Oetting W.S., Mentink M.M., Summers C.G., A Lewis R., White J.G., A King R. (1991). Three different frameshift mutations of the tyrosinase gene in type IA oculocutaneous albinism. Am. J. Hum. Genet..

[18] Marti A., Lasseaux E., Ezzedine K., Léauté-Labrèze C., Boralevi F., Paya C., Coste V., Deroissart V., Arveiler B., Taieb A. (2017). Lessons of a day hospital: Comprehensive assessment of patients with albinism in a European setting. Pigment Cell Melanoma Res..

[19] Breimer L.H., Winder A.F., Jay B., Jay M. (1994). Initiation codon mutation of the tyrosinase gene as a cause of human albinism. Clin. Chim. Acta.

[20] Kars M.E., Başak A.N., Onat O.E., Bilguvar K., Choi J., Itan Y., Çağlar C., Palvadeau R., Casanova J.-L., Cooper D.N. (2021). The genetic structure of the Turkish population reveals high levels of variation and admixture. Proc. Natl. Acad. Sci. USA.

[21] Jansonius N.M., Van Der Vliet T.M., Cornelissen F.W., Pott J.W.R., Kooijman A.C. (2001). A Girl Without a Chiasm: Electrophysiologic and MRI Evidence for the Absence of Crossing Optic Nerve Fibers in a Girl With a Congenital Nystagmus. J. Neuro-Ophthalmol..

[22] Apkarian P., Tijssen R. (1992). Detection and maturation of VEP albino asymmetry: An overview and a longitudinal study from birth to 54 weeks. Behav. Brain Res..

[23] Russell-Eggitt I., Kriss A., Taylor D.S. (1990). Albinism in childhood: A flash VEP and ERG study. Br. J. Ophthalmol..

[24] Kruijt C.C., de Wit G.C., Talsma H.E., Schalij-Delfos N.E., van Genderen M.M. (2019). The Detection Of Misrouting In Albinism: Evaluation of Different VEP Procedures in a Heterogeneous Cohort. Investig. Opthalmol. Vis. Sci..

[25] Creel D.J., Summers C.G., King R.A. (1990). Visual anomalies associated with albinism. Ophthalmic Paediatr. Genet..

[26] Pott J., Jansonius N., Kooijman A. (2003). Chiasmal coefficient of flash and pattern visual evoked potentials for detection of chiasmal misrouting in albinism. Doc. Ophthalmol..

[27] Schmitz B., Schaefer T., Krick C.M., Reith W., Backens M., Kasmann-Kellner B. (2003). Configuration of the Optic Chiasm in Humans with Albinism as Revealed by Magnetic Resonance Imaging. Investig. Opthalmol. Vis. Sci..

[28] Tomita Y., Takeda A., Okinaga S., Tagami H., Shibahara S. (1989). Human oculocutaneous albinism caused by single base insertion in the tyrosinase gene. Biochem. Biophys. Res. Commun..

[29] Kubal A., Dagnelie G., Goldberg M. (2009). Ocular albinism with absent foveal pits but without nystagmus, photophobia, or severely reduced vision. J. Am. Assoc. Pediatr. Ophthalmol. Strabismus.

[30] Xu T., Zhou Q., Li Y., Bai Y., Zhang W. (2020). Novel compound heterozygous variants of tyrosinase gene in an isolated foveal hypoplasia patient without nystagmus. J. Hum. Genet..

[31] Al-Araimi M., Pal B., Poulter J.A., Van Genderen M.M., Carr I., Cudrnak T., Brown L., Sheridan E., Mohamed M.D., Bradbury J. (2013). A new recessively inherited disorder composed of foveal hypoplasia, optic nerve decussation defects and anterior segment dysgenesis maps to chromosome 16q23.3-24.1. Mol. Vis..

[32] Thomas M.G., Kumar A., Mohammad S., Proudlock F.A., Engle E.C., Andrews C., Chan W.-M., Thomas S., Gottlob I. (2011). Structural Grading of Foveal Hypoplasia Using Spectral-Domain Optical Coherence Tomography: A Predictor of Visual Acuity?. Ophthalmology.

[33] McEntagart M., Williamson K.A., Rainger J.K., Wheeler A., Seawright A., De Baere E., Verdin H., Bergendahl L.T., Quigley A., Rainger J. (2016). A Restricted Repertoire of De Novo Mutations in ITPR1 Cause Gillespie Syndrome with Evidence for Dominant-Negative Effect. Am. J. Hum. Genet..

[34] Thorvaldsdóttir H., Robinson J.T., Mesirov J.P. (2013). Integrative Genomics Viewer (IGV): High-performance genomics data visualization and exploration. Brief. Bioinform..

[35] McKenna A., Hanna M., Banks E., Sivachenko A., Cibulskis K., Kernytsky A., Garimella K., Altshuler D., Gabriel S., Daly M. (2010). The Genome Analysis Toolkit: A MapReduce framework for analyzing next-generation DNA sequencing data. Genome Res..

[36] Li H., Handsaker B., Wysoker A., Fennell T., Ruan J., Homer N., Marth G., Abecasis G., Durbin R. (2009). 1000 Genome Project Data Processing Subgroup. The Sequence Alignment/Map format and SAMtools. Bioinformatics.

[37] Wang K., Li M., Hakonarson H. (2010). ANNOVAR: Functional annotation of genetic variants from high-throughput sequencing data. Nucleic Acids Res..

[38] Landini S., Mazzinghi B., Becherucci F., Allinovi M., Provenzano A., Palazzo V., Ravaglia F., Artuso R., Bosi E., Stagi S. (2019). Reverse Phenotyping after Whole-Exome Sequencing in Steroid-Resistant Nephrotic Syndrome. Clin. J. Am. Soc. Nephrol..

[39] Richards S., Aziz N., Bale S., Bick D., Das S., Gastier-Foster J., Grody W.W., Hegde M., Lyon E., Spector E. (2015). Standards and guidelines for the interpretation of sequence variants: A joint consensus recommendation of the American College of Medical Genetics and Genomics and the Association for Molecular Pathology. Genet. Med..

