# Supplementary material for: Expanding the Spectrum of Oculocutaneous Albinism: Does Isolated Foveal Hypoplasia Really Exist?
Source: Int J Mol Sci. 2022 Jul 15;23(14):7825. doi: 10.3390/ijms23147825 (PMC9317384; doi:10.3390/ijms23147825)
Supplement: Supplementary file 1 [file ijms-23-07825-s001.zip › ijms-1749033-supplementary.pdf]

Table S1. Detailed microsatellite markers. I: informative; NI: not informative.

**Microsatellite markers**	**Allele Size (bp)**				**Comments**
	Father	Mother	Proband 4	Proband 5	
**D11S4197**	250-260	252-260	250-252	250-252	I
**D11S1887**	257-265	262-262	257-262	257-262	I
**D11S4950**	132-132	132-132	132-132	132-132	NI
**D11S1780**	167-177	177-177	177-177	177-177	I
**D11S1367**	218-222	218-230	222-218	222-218	I
**BV088065**	177-185	177-181	177-181	177-181	I
**D11S4175**	172-184	144-180	172-144	172-144	I
**D11S931**	247-257	247-262	257-262	257-262	I
**D11S1358**	115-118	115-115	118-115	118-115	I
